# Disappearance of SARS-CoV-2 Antibodies in Infants Born to Women with COVID-19, Wuhan, China

**DOI:** 10.3201/eid2610.202328

**Published:** 2020-10

**Authors:** Jinzhi Gao, Wei Li, Xiaolin Hu, Ying Wei, Jianli Wu, Xiaoping Luo, Suhua Chen, Ling Chen

**Affiliations:** Tongji Hospital, Tongji Medical College, Huazhong University of Science and Technology, Wuhan, China

**Keywords:** COVID-19, 2019 novel coronavirus disease, SARS-CoV-2, severe acute respiratory syndrome coronavirus 2, viruses, respiratory infections, zoonoses, antibody, infant, passive immunity, vertical transmission, Wuhan, China

## Abstract

We report the detection and decline over time of severe acute respiratory syndrome coronavirus 2 antibodies in infants born to women with coronavirus disease. Among 11 infants tested at birth, all had detectable IgG and 5 had detectable IgM. IgG titers with positive IgM declined more slowly than those without.

Although the diagnosis of coronavirus disease (COVID-19) by reverse transcription PCR (RT-PCR) is efficient and specific, IgM and IgG production and decay are useful to assess past or recent infection, especially for patients with negative nucleic acid tests (*1*). Evidence of IgM and IgG in adults with COVID-19 appeared around 13 days after illness onset (*2*). Plateau IgM levels lasted for 4 weeks and gradually declined (*3*). Although IgG lasted for a longer time, only 19.5% patients had a >4-fold increase in titers during convalescence, a finding that was helpful for diagnosis of existing or acute infection (*2*,*3*).

However, to our knowledge, antibody persistence in infants born to women with COVID-19 has not yet been reported. IgM is the antibody isotype produced initially in the immune response and the first immunoglobulin class to be synthesized by a fetus or infant. Maternal IgM does not cross the placental barrier intact; therefore, positive IgM in early infants is potential evidence of intrauterine vertical transmission (*1*). Although IgG is transferred passively from mother to fetus through the placenta, the duration of passive immunity from maternal IgG is still unclear. 

We implemented assays for severe acute respiratory syndrome coronavirus 2 (SARS-CoV-2)–specific antibodies and SARS-CoV-2 nucleic acid tests in 64 infants admitted to the neonatal section of Tongji Hospital (Wuhan, China) during January 19–April 12, 2020. Among these, 24 infants (ranging in gestational age from 31 weeks to 41 weeks, 2 days) were born to women with PCR-confirmed COVID-19 (Table) and 40 infants (ranging in gestational age from 35 weeks, 3 days, to 41 weeks, 3 days) were born to women without COVID-19. Because antibody testing was implemented in early March, the timing of antibody testing in infants was inconsistent. We conducted SARS-CoV-2 nucleic acid tests by using a qualitative SARS-CoV-2 RT-PCR (DAAn GENE Biotech, http://www.daangene.com). We performed quantitative assessment of IgG and IgM by using the IFlash3000 Chemiluminescence Immunoassay Analyzer (YHLO Biotech, http://en.szyhlo.com), which has been proven to be a highly accurate method to detect SARS-CoV-2 antibodies (*4*). We considered IgM or IgG titers >10 AU/mL to be positive.

**Table:** Sequential severe acute respiratory syndrome coronavirus 2–specific antibodies assay in 24 infants born to mothers with PCR-confirmed coronavirus disease, Wuhan, China, January 19–April 12, 2020*

Case no.										GA, wk+d	
										At symptom onset	At birth
		IgM/IgG titers, AU/mL, by age of infant									
		0 d	7 d	14 d	28 d	45 d	60 d	75 d			
1	Mother							7.5/116.9*		33+4	36+3
	Infant							0.8/0.3			
2	Mother							4.4/**118.3**		38+5	38+5
	Infant							0.5/2.5			
3	Mother						5.0/**83.3**			37+5	38+2
	Infant						0.4/0.2				
4	Mother					**14.5/100.8**				39+1	39+5
	Infant					0.3/0.3					
5	Mother									34	35+2
	Infant		0.7/2.8	0.4/3.5	0.3/0.9						
6	Mother					7.0/**93.0**				39+3	40
	Infant					0.4/0.3					
7	Mother					**51.1/94.6**				39+4	41+2
	Infant					0.3/0.9					
8	Mother						**67.0/92.6**			37+4	38+4
	Infant						0.3/0.1				
9	Mother					**17.8/104.3**				38+2	39+5
	Infant					0.4/8.2					
10	Mother				**15.3/95.4**			9.9/**108.7**		38+1	39+5
	Infant				2.2/**32.7**			0.7/**19.5**			
11	Mother				**11.3/112.5**			5.3/**105.6**		35+5	38+2
	Infant		3.6/**166.0**	5.3/**120.8**	1.0/**36.2**			0.4/**16.2**			
12	Mother					**+/+†**		**37.9/94.6**		29+4	31
	Infant		5.7/**142.0**	2.6/**113.9**	1.8/**75.3**	0.3/**22.0**		0.4/**11.3**			
13	Mother				**+/+**					34+3	38
	Infant	3.5/**134.1**	2.4/**125.0**	2.8/**119.0**	0.7/**24.6**						
14	Mother				7.0/**61.1**		1.9/**59.5**			34+6	39
	Infant	2.2/**78.4**	1.5/**76.9**	3.0/**80.9**	0.8/**28.6**		0.5/**13.0**				
15	Mother	0.7/**58.9**								NA	37+2
	Infant	8.5/**174.5**			7.8/**122.7**						
16	Mother	**+/+**								NA	38+3
	Infant	0.7/**101.0**									
17	Mother	**+/+**				1.5/**104.2**				NA	38+3
	Infant	1.2/**110.4**	**10.4/108.9**		2.1/**68.2**						
18	Mother	–**/+**			4.8/**54.5**					NA	39+5
	Infant	6.6/**96.2**	**11.3/86.0**	**11.9/88.3**	2.5/**60.9**						
19	Mother	**2,581.6/281**			**40.2/89.8**	**13.9/99.6**		6.9/**94.2**		32+1	38
	Infant	**184.3/147.2**	**42.6/161.6**	**21.2/83.8**	5.5/**79.2**	1.6/**57.0**		0.3/**25.9**			
20	Mother			6.9/**82.8**			6.0/**81.3**			31+1	39+5
	Infant			**25.7/68.8**		3.0/**58.4**	0.4/**47.4**				
21	Mother	**+/+**					5.1/**139.0**			30+3	38
	Infant	**26.0/145.8**	**41.8/120.1**	**42.9/110.1**	**38.0/89.5**		**14.2/105.3**				
22	Mother	**+/+**								30	30
	Infant	**22.8/116.5**	**11.8/104.4**	8.7/**99.8**	1.9/**75.2**						
23	Mother	**+/+**			**10.5/101.6**			4.3/**115.6**		32+1	32+1
	Infant	**15.8/222.1**	**33.8/182.5**	9.0/**88.2**	6.1/**82.5**			0.5/**31.1**			
24	Mother	**+/+**	–**/+**		8.0/**83.9**					NA	NA
	Infant	**30.1/106.0**	**29.5/84.6**	**31.5/94.9**	9.7/**73.1**						

*Bold text indicates titer >10 AU/mL (i.e., above cutoff). GA, gestational age; NA, not available; –, negative; +, positive. †Some mothers delivered in other hospitals, and antibody testing was conducted using other qualitative antibody-detection kits.

Among the 40 infants born to women without COVID-19, results of nucleic acid tests from throat and anal swab specimens and results of antibody assays were negative. Among the 24 infants born to women with COVID-19, 15 (62.5%) had detectable IgG and 6 (25.0%) had detectable IgM; nucleic acid test results were all negative. None of the 24 infants had complications related to pneumonia, a finding that is consistent with a previous report (*5*). Among 11 infants with antibody titers detected at birth, all had detectable IgG (100%) and 5 (45.5%) had detectable IgM, 1 of whom had high IgM levels (infant described in case 19 in the Table). Although the IgG titers in all 15 infants with positive IgG decreased gradually, the IgG levels declined more slowly in infants with positive IgM compared with those without (Figure). The infant described in case 19 was born 33 days after the mother had COVID-19 diagnosed (41 days since symptom onset) and by elective cesarean section at 38 weeks’ gestation because of the mother’s previous cesarean section. The infant had high IgM and IgG titers in the umbilical cord blood and peripheral blood on the first day of life, which gradually decreased at repeated tests thereafter (Table). The infant also had negative nucleic acid tests results in a series of specimens, including cord blood, placenta, amniotic fluid, stool, urine, peripheral blood, and gastric juice at different timepoints. The placenta sample described in case 19 was collected during surgery and sent for pathologic examination, which revealed slight inflammation with slight fibrin deposition and lymphocyte infiltrates (Appendix Figure).

**Figure:**
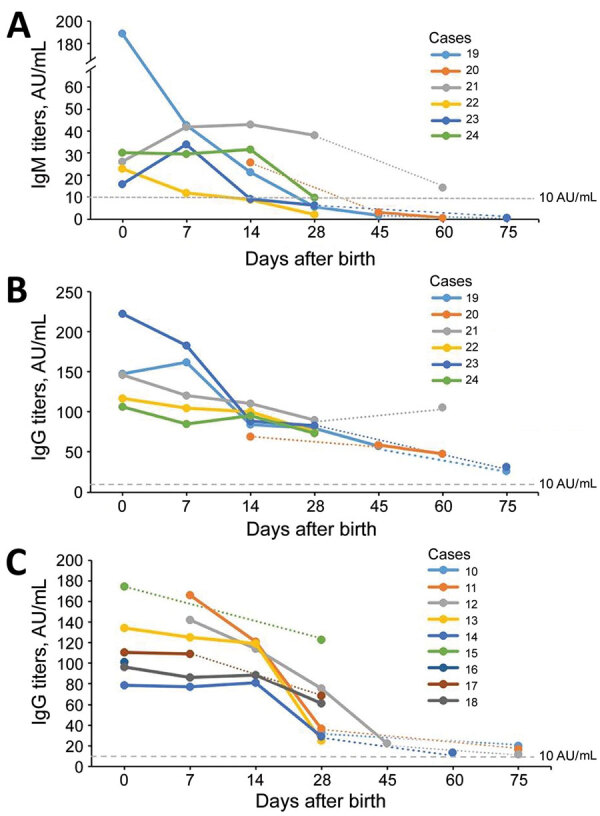
Temporal changes in severe acute respiratory syndrome coronavirus 2–specific antibodies in infants born to women with coronavirus disease, Wuhan, China. A, B) Dynamic changes of IgM (A) and IgG (B) titers in infants with positive IgM. C) Dynamic changes of IgG titers in infants with negative IgM. The IgM and IgG titers gradually decreased with time. IgG titers with positive IgM declined more slowly than those without, and the duration was as long as 75 days.

Although positive results in SARS-CoV-2 nucleic acid or virus-specific IgM in infants have been reported previously, evidence of vertical transmission of SARS-CoV-2 is not complete (*6*–*8*). We report the dynamic changes of SARS-CoV-2–specific antibodies in infants born to mothers with COVID-19. Five of 11 infants were seropositive for IgM at birth; however, these findings was not sufficient to confirm SARS-CoV-2 vertical transmission without positive nucleic acid testing. The study was also limited by a small sample size. However, these findings show a rapid rate of decline in antibody titers, suggesting lack of protective passive immunity in infants, and IgM detection in infants, supporting a growing body of evidence of possible vertical transmission. We still do not have a correlate of immunity (e.g., we do not know exactly what level of antibody titers are considered protective against infection), and whether infants testing positive by PCR at birth have higher levels of IgM or IgG remains to be seen. More work is needed to understand SARS-CoV-2 immunity in infants; such findings might have implications for potential vaccination efforts.

Appendix Additional information about disappearance of SARS-CoV-2 antibodies in infants born to women with COVID-19, Wuhan, China.

## References

[1] Kimberlin DW, Stagno S. Can SARS-CoV-2 infection be acquired in utero? More definitive evidence is needed. JAMA. 2020; Epub ahead of print. 10.1001/jama.2020.4868 32215579

[2] Long QX, Liu BZ, Deng HJ, Wu GC, Deng K, Chen YK, et al. Antibody responses to SARS-CoV-2 in patients with COVID-19. Nat Med. 2020;26:845–8. 10.1038/s41591-020-0897-1 32350462

[3] Xiao AT, Gao C, Zhang S. Profile of specific antibodies to SARS-CoV-2: The first report. J Infect. 2020;81:147–78. 10.1016/j.jinf.2020.03.012 32209385 PMC7118534

[4] Infantino M, Grossi V, Lari B, Bambi R, Perri A, Manneschi M, et al. Diagnostic accuracy of an automated chemiluminescent immunoassay for anti-SARS-CoV-2 IgM and IgG antibodies: an Italian experience. J Med Virol. 2020;jmv.25932; Epub ahead of print. 10.1002/jmv.25932 32330291 PMC7264663

[5] Liu W, Wang J, Li W, Zhou Z, Liu S, Rong Z. Clinical characteristics of 19 neonates born to mothers with COVID-19. Front Med. 2020;14:193–8. 10.1007/s11684-020-0772-y 32285380 PMC7152620

[6] Alzamora MC, Paredes T, Caceres D, Webb CM, Valdez LM, La Rosa M. Severe COVID-19 during pregnancy and possible vertical transmission. Am J Perinatol. 2020;37:861–5; Epub ahead of print. 10.1055/s-0040-1710050 32305046 PMC7356080

[7] Zeng H, Xu C, Fan J, Tang Y, Deng Q, Zhang W, et al. Antibodies in infants born to mothers with COVID-19 pneumonia. JAMA. 2020;323:1848. 10.1001/jama.2020.4861 32215589 PMC7099444

[8] Dong L, Tian J, He S, Zhu C, Wang J, Liu C, et al. Possible vertical transmission of SARS-CoV-2 from an infected mother to her newborn. JAMA. 2020;323:1846–8. 10.1001/jama.2020.4621 32215581 PMC7099527

