# Supplementary material for: Disappearance of SARS-CoV-2 Antibodies in Infants Born to Women with COVID-19, Wuhan, China
Source: Emerg Infect Dis. 2020 Oct;26(10):2491–4. doi: 10.3201/eid2610.202328 (PMC7510696; doi:10.3201/eid2610.202328)
Supplement: Appendix — Additional information about disappearance of SARS-CoV-2 antibodies in infants born to women with COVID-19, Wuhan, China. [file 20-2328-Techapp-s1.pdf]

Article DOI: https://doi.org/10.3201/eid2610.202328

Disappearance of SARS-CoV-2 Antibodies in Infants Born to Women with COVID-19, Wuhan, China

Appendix

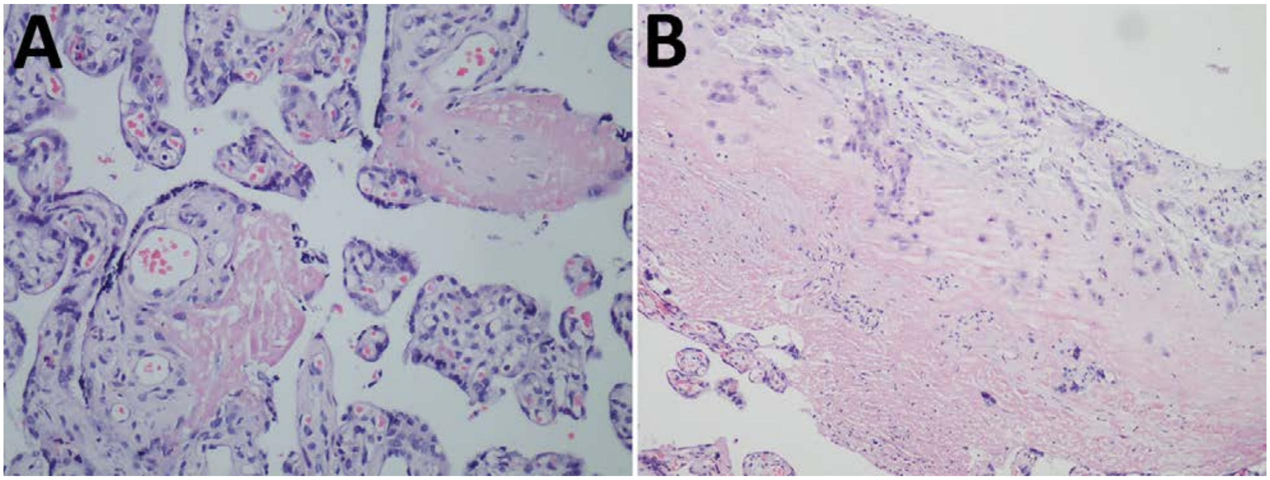

Appendix Figure. Pathologic examination of the placenta. A) Normal placenta villi. B) Slight fibrin deposition and lymphocytes infiltrate.
